# A Dental Handbook on Surgery

**Published:** 1913-09-15

**Authors:** 


### BIBLIOGRAPHY.

A Dental Handbook on Surgery: There has just appeared a most excellent little work on the general principles of surgery adapted to the needs of the dentist. It is written by Arthur S. and Bayford Underwood of London, Eng. The work covers the field of general surgery with the exception of the administration of anesthetics and the special field of surgery in which the dentist can have little or no interest. The treatment of each subject is necessarily brief but is very cleverly stated. We have seldom seen a brief work of so comprehensive scope that was so satisfactorily done. It is a book that every dentist ought to have in his library and we are sure he will practice his profession with more intelligence after reading this book. It is published in this country by Wm. Wood and Co. of New York, and may be had through the dental supply houses.

